# Advances in Skin Tissue Bioengineering and the Challenges of Clinical Translation

**DOI:** 10.3389/fsurg.2021.640879

**Published:** 2021-08-24

**Authors:** Bronwyn L. Dearman, Steven T. Boyce, John E. Greenwood

**Affiliations:** ^1^Skin Engineering Laboratory, Adult Burns Centre, Royal Adelaide Hospital, Adelaide, SA, Australia; ^2^Adult Burns Centre, Royal Adelaide Hospital, Adelaide, SA, Australia; ^3^Faculty of Health and Medical Science, The University of Adelaide, Adelaide, SA, Australia; ^4^Department of Surgery, University of Cincinnati, Cincinnati, OH, United States

**Keywords:** skin, bioengineering, burns, wound closure, skin substitutes, clinical translation, tissue engineering, biopolymers

## Abstract

Skin tissue bioengineering is an emerging field that brings together interdisciplinary teams to promote successful translation to clinical care. Extensive deep tissue injuries, such as large burns and other major skin loss conditions, are medical indications where bioengineered skin substitutes (that restore both dermal and epidermal tissues) are being studied as alternatives. These may not only reduce mortality but also lessen morbidity to improve quality of life and functional outcome compared with the current standards of care. A common objective of dermal-epidermal therapies is to reduce the time required to accomplish stable closure of wounds with minimal scar in patients with insufficient donor sites for autologous split-thickness skin grafts. However, no commercially-available product has yet fully satisfied this objective. Tissue engineered skin may include cells, biopolymer scaffolds and drugs, and requires regulatory review to demonstrate safety and efficacy. They must be scalable for manufacturing and distribution. The advancement of technology and the introduction of bioreactors and bio-printing for skin tissue engineering may facilitate clinical products' availability. This mini-review elucidates the reasons for the few available commercial skin substitutes. In addition, it provides insights into the challenges faced by surgeons and scientists to develop new therapies and deliver the results of translational research to improve patient care.

## Introduction

The challenges of translational medicine are becoming more prevalent with developing new technologies as novel therapies for personalised medicine. One therapy where translational research is at the forefront is reducing the use of skin autografts for extensive full-thickness burns with laboratory-generated skin (1–7). The split-thickness meshed and expanded skin autograft has been the prevailing standard of care for burns surgeons for decades and remains the preferred method of wound closure due to its relatively high efficacy of stable wound closure (3, 8, 9). However, if the burn area massively exceeds the area of available donor site for skin autografts, the advantages of autologous engineered skin substitutes is compelling. To regenerate a substitute of uninjured human skin that definitively provides wound closure both anatomically and physiologically (6) is a common challenge for tissue engineers, and may involve polymer chemists, cellular and molecular biologists, surgeons, nurses, and therapists. A systematic review of clinical studies investigating autologous bilayered skin substitutes as epithelial stem cell niches after grafting, identified 16 potential studies and nine types of autologous skin substitutes over a 25-year period (10). Currently, only a small number of these are still available for therapeutic use, with no ideal substitute in the market. The current models have distinct attributes, for the majority, the scaffold type is a source or derivation of collagen (biologic) with autologous fibroblasts and keratinocytes. Another novel synthetic scaffold utilising a polyurethane (PUR) has also been used to generate a skin composite (composite cultured skin, CCS) and has reported its use for the treatment of a 95% total body surface area burn patient (11). However, these all remain deficient in pigmentation, hair, and other dermal appendages. The authors draw on combined experiences from taking the research bench to bedside. This review will describe the distinct models of bilayered tissue engineered products that have been used therapeutically, which there are few, but all address the same clinical challenges.

### The Need for an Alternative to Skin Autografts for Extensive Full-Thickness Burns

Burns are a global health concern, especially for low to middle-income countries, accounting for over 95% of burn deaths (12). Burn injuries of all depths make up only a small proportion (1%) of trauma hospitalisations in Australia (13), but are one of the most costly, due to long hospital and rehabilitation stays (14, 15). In the United States, hospitalised burns cost over $1billion per year (16) and in high income countries the mean cost per 1% TBSA is US$4159.00 (17). These costs are significant, but the major indirect cost is the patient's lifelong scars and disfigurements. As the percentage of total body surface area (TBSA) burn and burn depth increases, the costs increase exponentially (14). Extensive, full-thickness burn injuries (>50% TBSA) usually require intensive care, multiple surgical procedures, physical and occupational therapy and psycho-social interventions to recover. Patients with these degrees of burn often die which obviates skin graft paucity (18). However, advances in burn care have led to increased survival rates due to early excision of eschar, temporary wound closure, advanced nutritional support, infection prevention, and improvements in critical care medicine (19–22). Although, burns in the elderly and those with coincident trauma such as inhalation injury, remain challenging.

In this patient population, temporary wound coverage provides time for utilisation of donor sites from superficially burned skin and re-harvesting to allow multiple procedures of skin autografting (23). Maximising wound coverage with available donor site involves thin, widely-meshed, or expanded (Meek-Wall technique) (24) skin autografts, resulting in poor functional and aesthetic outcomes. In addition, donor sites generated by split-thickness skin graft harvesting are extremely painful, may require opiate analgesia, limit mobilisation, and discourage compliance with physical therapy (25). Skin autografts, however, have properties that promote their continued widespread use for the closure of large, deep skin wounds (no rejection, vascular inosculation, high efficacy, long-term stability), but their correlation with increased morbidity, especially in the elderly, is a significant disadvantage, which motivates the search for alternatives (26, 27).

An ideal skin substitute should adhere, vascularise, and integrate quickly, contain both epidermal and dermal components, provide permanent and definitive wound closure, be autologous, resist infection, be easy to prepare, handle well, easy to apply, cost-effective, and resist mechanical shear forces (28). They should demonstrate high engraftment rates, restore natural pigmentation, and provide all skin appendages and sensory networks in uninjured skin. This list of qualities is comprehensive, and to simultaneously replicate these features *in vivo* requires complex engineering in the laboratory. Engineered skin fabrication is a specialised professional field with many aspects still to be elucidated and reduced to practise. A standardised universal classification system for “skin substitutes” was published by Davison-Kotler et al. in 2018 to encapsulate all adaptations (research and clinical) using a factorial design (29). Primary categories include acellular dermal substitutes, temporary skin substitutes, and permanent skin substitutes, further expanding into sub-categories (2). The many variations have been tabulated in former reviews and will not be detailed here (6, 7, 29–38). This review focuses on permanent, cellular, and mainly autologous products with dermal and epidermal components. It will explore a few commercially available products and some clinically used in extensive wounds (Table 1).

**Table 1:** Examples of clinically-available or investigative skin substitutes [adapted from Vig et al. (7), Boyce et al. (31)].

	**Product-country of origin**	**Classification/components**	**Proposed clinical indication**	**Product limitations**
Dermal-epidermal substitutes	TISSUEtech Autograft system (Hylomatrix + Laserskin)—Italy (39, 40)	Cellular, autologous Ks, Fbs with HA	Diabetic ulcers	Small wounds, difficult to use clinically
	Tissue-cultured skin autograft (TCSA's)—Germany (41)	Cellular, autologous Ks, Fbs with Matriderm™	Chronic ulcerations	Small wounds
	Engineered skin substitute (ESS)—USA (42–50)	Cellular, autologous Ks, Fbs, bovine collagen-GAG	Large burns and other skin loss conditions	Xenogeneic scaffold, small pieces, shrinkage of product, cost
	Composite cultured skin (CCS)—Australia (51–57)	Cellular, autologous Ks, Fbs, synthetic polymer	Full- thickness burns	Scaffold porosity, complex
	Self-assembled skin substitute (SASSs)—Canada (58)	Cellular, autologous Ks and Fbs	Burns	Labour intensive
	Autologous homologous skin construct (AHSC)—USA (59–61)	Cellular, autologous skin cells	Burns, acute trauma chronic wounds	Cell suspension/aggregate
	MyDerm—Malaysia (62–65)	Cellular, autologous Ks and Fbs, Fibrin	Burns, skin trauma and chronic wounds	Clinical efficiency
	StrataGraft™–USA (66, 67)	Cellular, allogeneic Ks and Fbs, non-bovine collagen	Burns and skin conditions	Allogeneic, temporary, size
	denovoSkin—Switzerland (68)	Cellular, autologous Ks and Fbs, bovine collagen	Burns	Xenogeneic scaffold

*HA, Hyaluronic acid; Ks, Keratinocytes, Fbs, Fibroblasts*.

### Large, Excised Wounds—Temporising the Wound Bed for Definitive Closure

The loss of the epidermis, and sufficient dermis to ensure loss of all the epidermal adnexa, requires rapid wound closure. The primary focus is on reducing inflammation and granulation, preventing infection and limiting contraction. The associated mortality and morbidity rates decrease with the successful implementation of the above and achieved by staged closure (69). Acellular dermal substitutes comprising a dermal and a pseudo-epidermal component have been widely used to achieve physiological closure. Their implementation has produced a paradigm shift in burn care (21, 70, 71). The dermal components may originate from decellularised human skin, biological polymers, or synthetic polymers. Their function is to temporarily close the excised wound to decrease fluid loss, allow integration and controlled granulation tissue invasion inducing a vascularised wound bed. Commercial examples include Integra® Dermal Regeneration Template, Pelnac, Terudermis, Hyalomatrix, and RenoSkin (69). These products and similar ones have limitations, including a risk of transmissible disease, loss from infection and high costs (5, 72). Despite regulatory approval for specific medical indications, most dermal substitutes have not achieved worldwide consensus as market leaders for large, deep dermal wounds. However, establishing a neo-dermis enhances structural stability and provides the time required for definitive epithelial wound closure, whether by serial grafting or by generating and applying autologous engineered skin.

The NovoSorb™ Biodegradable Temporising Matrix (BTM) is a synthetic scaffold that is currently in routine use for burns and complex wound repair (73–79). It is a scaffold that temporises the wound and biodegrades after integration and establishment of dermal elements (80). Furthermore, it resists infection, can be made in large sheets, is inexpensive to produce, easy to handle, and provides integration time (76–82). With the optimisation of a dermal replacement template and a major limitation addressed, i.e., acquisition of time for cellular growth, the prospective next step is the specification of a definitive wound closure alternative.

### The Current State of Bioengineered Dermal-Epidermal Substitutes

Bioengineered skin substitutes involving dermal and epidermal components are the focus of this paper; however, epidermal replacements (cellular) require brief reference to appreciate the desirability of both components. A skin substitute is yet to be achieved that replaces the anatomy and physiology of uninjured skin or completely replaces all skin autograft properties- implying why an epidermal replacement alone will not replicate a meshed, or sheet, autograft. Cultured Epithelial Autografts (CEA's) have been used since 1986 (83), and other adaptations or iterations of keratinocyte suspensions [e.g., Epicel (84, 85), Cell Spray, RECELL® (86), BioSeed (87), Laserskin (39, 40)] have evolved. These are clinical adjuncts to therapies with traditional treatments of burn care to expedite reepithelialisation rate. Clinically applicable for small wounds (88), ulcers (87, 89–92), superficial burns (93) and skin graft donor sites; they have not been universally accepted by burns surgeons independently for deep large burns due to their limited expansion rate, mechanical fragility on handling, tendency to blister *in vivo* and vulnerability to shear after application (partly to deficiencies in basement membrane formation) (94). In addition, they are costly to produce, can take weeks to manufacture, and are epidermal derived replacements (95–100). Incorporating a substitute containing epidermal and dermal components is a logical progression toward regenerating a tissue more like uninjured skin (101).

A critical paracrine dialogue between fibroblasts and keratinocytes is essential for basement membrane synthesis, a beneficial feature for engineered skin substitutes (102–104). The basement membrane protects against shear by establishing a molecular bond that anchors the cellular epidermis to the extracellular matrix of the dermis. The most analogous to skin, and the most successful clinically to date, is an Engineered Skin Substitute (ESS) developed in Cincinnati, Ohio (105). Developed over the past 30 years, the ESS comprises autologous keratinocytes and fibroblasts in a bovine collagen-glycosaminoglycan (GAG) scaffold (42–50). The ESS model was the first to demonstrate stable closure of full-thickness burns by combination with Integra® Dermal Regeneration Template (106). In 2017, a report was published of ESS' clinical results in 16 subjects treated from 2007 to 2010. For patients with >50%, TBSA full-thickness burns, ESS's were able to reduce the need for harvesting donor skin grafts and reduce the mortality rate compared with data from similar patient populations reported in the National Burn Repository of the American Burn Association (107). The ESS results in a closed wound that has structural and functional similarities to native skin. However, this model also has limitations (lack of other cell types and adnexal structures, contraction of the collagen scaffold during ESS fabrication, relatively high cost and regulatory complexity); and is not commercially available. Pre-clinical studies have recently demonstrated the successful incorporation of melanocytes (108, 109), microvascular endothelial cells (110), and hair follicles (111) into the ESS model.

Bovine collagen is also used in denovoSkin™ (Cutiss AG, Zurich), which consists of a collagen hydrogel and human dermal fibroblasts and keratinocytes. It has been classified as an Advanced Therapy Medical Product (ATMP) and has received FDA and EMA Orphan status to treat burns in the US and EU (112, 113). It is currently undergoing clinical trial recruitment for adult and children burns, with an estimated completion date of 2023. However, the production of a dermal-epidermal equivalent with xenogeneic (non-human)-derived biologicals, such as bovine, rat, or porcine collagens or glycosaminoglycans (42) raises the potential for immune recognition and rejection and risk of prion transmission. A synthetic scaffold and autologous cell approach may reduce these risks.

Several matrices using fibroblasts alone to provide the biological extracellular matrix environment (114–117) have shown the generated skin's long-term stability *in vitro* (118). Through the Special Access Program in Canada, a Self-Assembled Skin Substitute (SASS) has shown clinical effectiveness, reporting a case series of 14 severely burned subjects (58). This substitute contains autologous fibroblasts and keratinocytes, forming a human biopolymer fibroblast scaffold with subsequent keratinocyte seeding. The constraining factor for this type of substitute, like some others, is the production time, with an average of 9 weeks from the initial biopsy (58). In addition, the SASSs post-transplantation displayed visible junctions between applications, re-iterating the need for a sizeable sheet that can be generated and transplanted with fewer anaesthetics.

Improved scalability has now been reported using a biodegradable polyurethane (PUR) as the scaffold for a dermal-epidermal alternative, known as a composite cultured skin (CCS) (51–57). The attributes for an “ideal engineered skin,” as mentioned previously (119), formulated the premise of combining an engineered-epidermis to a modified BTM dermal substitute. Compared to bovine collagen (a biologic), a synthetic biodegradable PUR showed lower toxicity and cytotoxicity, reduced immunogenic reaction, and minimal inflammatory response (51, 52, 120). The BTM-CCS provided a two-stage strategy, with the CCS as a definitive second stage wound closure material. The application of NovoSorb™ BTM, a temporising matrix, addresses one of the major limitations of available skin substitutes [i.e., time required for autologous cell expansion 3–5 weeks (38)]. The integration period enables the time required for cell isolation, expansion and bilayered construction (up to 7-weeks if needed) (11, 69). The CCS is a 1 mm thick PUR porous scaffold, populated with autologous fibroblasts in a fibrin network and layered with autologous keratinocytes (53, 54). Pre-clinical studies in a porcine model initially demonstrated the efficacy of small CCS, and later large pieces, generated in an automated bioreactor (54, 57). This custom-made novel bioreactor device has taken this from research to clinic (11, 57). The two-stage strategy of BTM-CCS has been used clinically in a 95% TBSA burn injury (covering 40% TBSA of original burn) (11). The patient not only survived but, at 1.5 years post-injury, required minimal contracture release in areas where autografts were applied and none to the CCS-applied areas. The result for CCS was a smooth, supple aesthetic appearance with varying pigmentation from primary epithelial engraftment. No delineation between junctions of CCSs can be observed. ROM and SOSS scores were comparable to sheet graft, but favourable over 1:3 meshed STSG and Meek.

The subcutaneous layer (the deepest layer of skin) is absent in many investigational and clinical substitutes. Polarity TE, a US company, produces an Autologous Homologous Skin Construct (AHSC). They claim that functional full-thickness skin can be regenerated by obtaining a full-thickness biopsy with immediate application (59). A retrospective, 15-patient post-AHSC application review case series was reported (60) for various wound types (burns, acute/traumatic injuries, and chronic wounds). It differs from the conventional dermal-epidermal substitute, in that it seems not to necessitate culture and is returned to the patient within days. These wounds were closed at 3 months post application; however, further studies are required to investigate and substantiate the claims of efficacy, especially in full-thickness, excised burns (61).

Several other dermal-epidermal constructs have been used clinically or gone to clinical trial pending commercialisation (Table 1). Some examples include, tissue cultured skin autograft (TCSAs using Matriderm™, Germany) (41), TISSUEtech Autograft system™ (using Hylomatrix, Anika Therapeutics Inc., Bedford) (121), and others using Allodermis (122, 123), Human plasma (124, 125), and Fibrin (MyDerm, Japan) (62–65). Another bi-layered product recently receiving (2021) FDA approval for adult deep partial-thickness burns is StrataGraft® (Mallinckrodt, USA) (66, 67). Although it is not autologous, this bilayered allogeneic product comprises murine collagen and allogeneic fibroblasts and keratinocytes, this acts indirectly on the autologous cells to assist with wound closure (66). This type of treatment is limited for deep full-thickness burns as it needs another source of autologous cells e.g., meshed graft or other skin appendages, to close the wound. However, it is readily available and “off-the-shelf” ready for immediate use, whereas typical bilayered autologous substitutes can take weeks to fabricate. As with any graft, there is potential for loss if there is no neovascularization. The majority of clinically available engineered skins are avascular; however, this is under investigation by researchers (126). The loss of graft can be due to an accumulation of blood (haematoma), fluid (seroma), contamination, or mechanical shear. The different skin models mentioned have varying pore sizes and can contribute to the success of the engraftment. The density of the dermal component (i.e., too small or large pores) can inhibit or promote vascularisation (57, 127). Shear of a substitute graft or blistering will also occur if there is loss or no basement membrane and reiterates the importance of cell-cell contact of the epidermal-dermal component *in vitro* culture. When this loss occurs, the wound heals by secondary reepithelialisation and healing is delayed. Although, a systematic review of bilayered skin substitutes showed wound healing rates for leg ulcers were comparable with the standard of care (RR 1.51, 95% 1.22–1.88) (128). A widely meshed STSG used for extensive wound coverage results in a weave-like pattern, producing a poor aesthetic result. In contrast, autologous engineered skin provides immediate coverage with a stratified epidermis that suppresses granulation tissue and arrests the scarring process. Producing a favourable smooth, pliable, even skin, with a reduction in pain and itch (55, 68, 107). Another major strength and benefit over skin autografts is the reduction of autologous donor skin and its associated morbidities. The diverse bilayered approaches mentioned all have their strengths and weaknesses, and in review, the ideal model may likely be combinations of biopolymer scaffolds and stem cells that can produce a functional, clinically safe and effective alternative (129, 130). Any of these tissue engineered products will face regulatory reviews and reimbursement requirements.

### Clinical Challenges for Skin Substitutes

As cell-based therapeutic inventions, these products require approval by regulatory authorities to ensure high quality, safety and proven efficacy (131) (Therapeutic Goods Administration, TGA in Australia; Food and Drug Administration, FDA in the United States; European Medicines Agency (EMA), in the European Union, etc.). Several pre-clinical substitutes are being used through Special Access Programs (SAP) in designated countries. This scheme is a way of using non-licenced products to treat life-threatening injuries where other methods are not suitable, or non-existent. In the United States, the passage of the 21st Century Cures Act, in 2016 (31) and new agency programs will facilitate the clinical use of novel products and devices to treat patients at severe medical risk.

**Figure 1:**
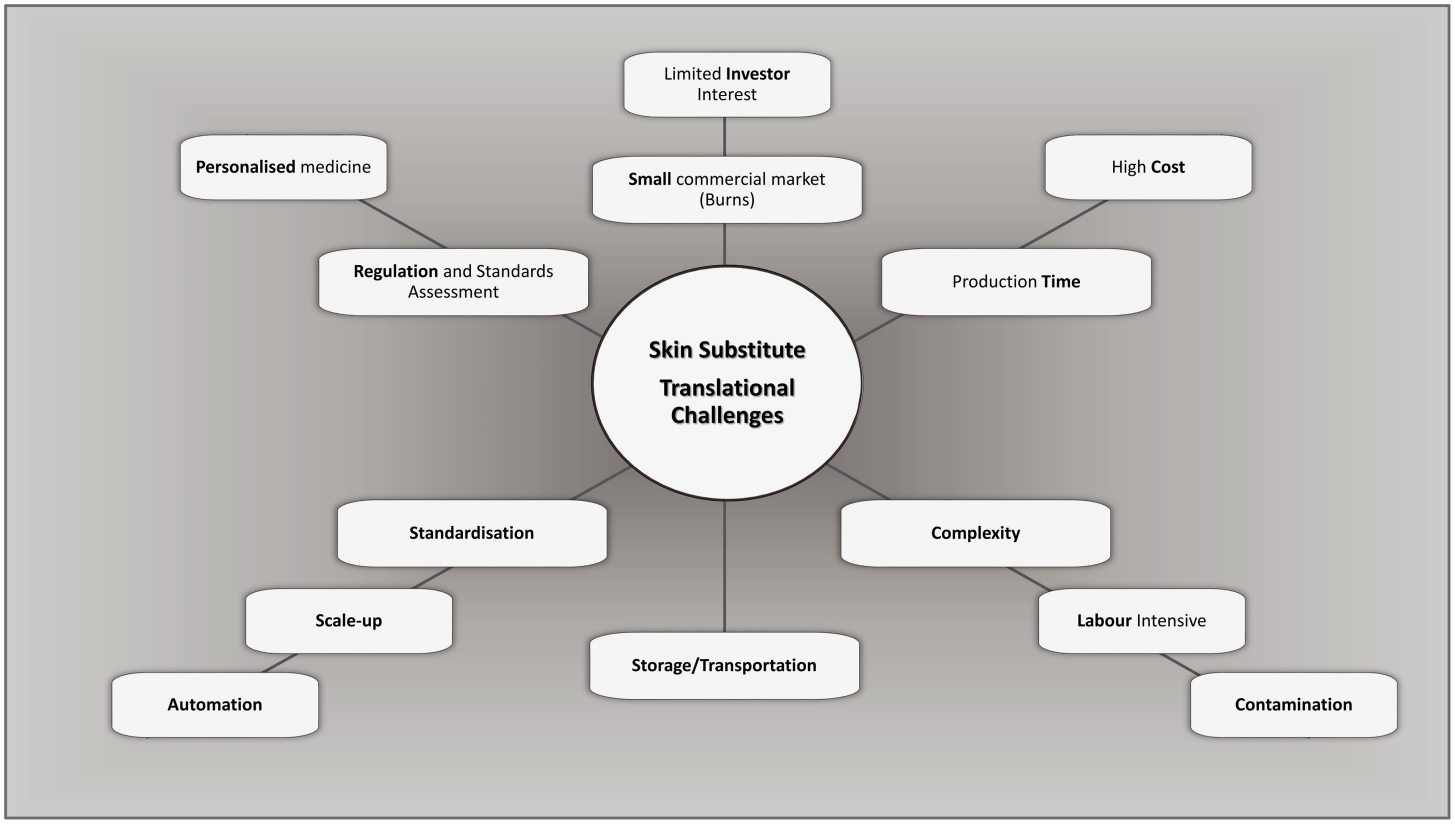
Challenges and considerations in bioengineering of bilayered skin substitutes. Adapted from Al-Himdani et al. (133).

The generation of highly manipulated tissue-engineered products follows the standards for current Good Manufacturing Practices (cGMP) (132). They should ideally be free of any xenogeneic product (131) and include mandatory testing for microbiological assessment [sterility assurance level (SAL) of 10^−6^] and transportation validation to ensure that product integrity is maintained. Generating a clinically viable, and ethical, product suitable for market is a lengthy and labour intensive process, with high initial capital costs. These infrastructure costs, process complexity, and stringent quality control result in expensive products, making commercialisation less practical (133, 134) and are translational challenges a therapeutic product may encounter (Figure 1). The cost of such substitutes, however, should not be assessed directly by the cost per unit of production only (31, 135), but also indirectly by assessing overall hospital cost reductions concerning length of stay, the number of reconstructive surgeries post-major burn, patient outcome and aesthetics. Although, an experienced highly trained medical team, including specialised nurses and therapy protocols are required during the intense early stages of treatment until they become the prevailing standard of care.

### The Future Opportunities of Skin Substitutes

The generation of laboratory-generated “skin substitutes,” irrespective of classification, have to date only partially addressed the requirements for achieving stable wound closure. They currently produce inadequate pigmentation (hypo- or hyperpigmentation), they lack vasculature, hair, glands, and none have replicated the results of unmeshed autograft or duplicated the anatomy and physiology of uninjured skin. Due to cost, regulatory restraints, and the significant scientific challenge to incorporate all skin features simultaneously (136–139). Approaches to the refinement of fabrication systems for skin substitutes will facilitate advanced models of engineered skin to reach their markets with a consequent decrease in costs. The requirement for scalability is a compelling demand for large burn injuries and can be met by incorporating automated bioreactors (57, 140). These may assist production and provide complete automation and standardisation to improve product quality. The robotic systems are engineering advances that will move forward in parallel with medical advances. The 3D and 4D bioprinting fields coupled with the latest compatible bioinks are novel techniques that may rapidly advance the tissue engineering field (141–145).

In time, these technologies and advances in tissue engineering will at least reduce, and possibly replace, the need for skin autografts and enable easier clinical translation of an acceptable autologous engineered skin, suitable for patient use. The significance of this is that patients with life-threatening burns will no longer suffer the painful acute morbidity and later scarring that donor sites generate. Time in ICU and total hospitalisation will be reduced, the need for reconstructive surgery will decrease, with overall costs reduced. The success will also have implications for other dermatologic conditions, including but not limited to giant congenital naevi excision and engraftment, epidermolysis bullosa treatment, certain surgical reconstructions, and vitiligo. It can also contribute to the investigation and requirement for epidermal appendages, naturally matched skin pigmentation, vascular plexus, and sensory nerves (2, 139, 146, 147). As each of these advances is currently under investigation, there can be high degrees of confidence that many, if not most of these skin components (uniform skin colour, sweat glands, and hair follicles) will be incorporated into future models of skin substitutes and available clinically for the treatment of full-thickness skin wounds, including burns.

## Author Contributions

BD developed the outline with contributions from both JG and SB. BD wrote the manuscript with editing by JG and SB.

## Conflict of Interest

At the time of the CCS studies JG was a major shareholder in PolyNovo Biomaterials Pty Ltd., the company that produced the NovoSorb^®^ Polyurethane material. JG and BD were stakeholders in Skin Tissue Engineering Pty Ltd. a company that had no protectable IP and was used as a vehicle for attracting and dispersing funding from non-grant awarding bodies. The remaining author declares that the research was conducted in the absence of any commercial or financial relationships that could be construed as a potential conflict of interest.

## Publisher's Note

All claims expressed in this article are solely those of the authors and do not necessarily represent those of their affiliated organizations, or those of the publisher, the editors and the reviewers. Any product that may be evaluated in this article, or claim that may be made by its manufacturer, is not guaranteed or endorsed by the publisher.
